# Temporal Summation in Fibromyalgia Patients: Comparing Phasic and Tonic Paradigms

**DOI:** 10.3389/fpain.2022.881543

**Published:** 2022-06-22

**Authors:** Luis Castelo-Branco, Alejandra Cardenas-Rojas, Ingrid Rebello-Sanchez, Kevin Pacheco-Barrios, Paulo S. de Melo, Paola Gonzalez-Mego, Anna Marduy, Karen Vasquez-Avila, Pablo Costa Cortez, Joao Parente, Paulo E. P. Teixeira, Gleysson Rosa, Kelly McInnis, Wolnei Caumo, Felipe Fregni

**Affiliations:** ^1^Neuromodulation Center and Center for Clinical Research Learning, Spaulding Rehabilitation Hospital and Massachusetts General Hospital, Harvard Medical School, Boston, MA, United States; ^2^Universidad San Ignacio de Loyola, Vicerrectorado de Investigación, Unidad de Investigación para la Generación y Síntesis de Evidencias en Salud, Lima, Peru; ^3^Instituto de Ciencias Biologicas, Departamento de Imunologia Basica e Aplicada, Manaus, Brazil; ^4^MGH Institute of Health Professions, Boston, MA, United States; ^5^Department of Physical Medicine and Rehabilitation, Spaulding Rehabilitation Hospital, Harvard Medical School, Boston, MA, United States; ^6^Pain and Palliative Care Service at Clinical Hospital of Porto Alegre (HCPA), Surgery Department, Federal University of Rio Grande Do Sul, Porto Alegre, Brazil

**Keywords:** temporal summation, TSPS, tonic, phasic, fibromyalgia, central sensitization, quantitative sensory testing, QST

## Abstract

**Introduction:**

Fibromyalgia (FM) is associated with dysfunctional pain modulation mechanisms, including central sensitization. Experimental pain measurements, such as temporal summation (TS), could serve as markers of central sensitization and have been previously studied in these patients, with conflicting results. Our objective in this study was to explore the relationships between two different protocols of TS (phasic and tonic) and test the associations between these measures and other clinical variables.

**Materials and Methods:**

In this cross-sectional analysis of a randomized clinical trial, patients were instructed to determine their pain-60 test temperature, then received one train of 15 repetitive heat stimuli and rated their pain after the 1st and 15th stimuli: TSPS-phasic was calculated as the difference between those. We also administered a tonic heat test stimulus at the same temperature continuously for 30 s and asked them to rate their pain levels after 10 s and 30 s, calculating TSPS-tonic as the difference between them. We also collected baseline demographic data and behavioral questionnaires assessing pain, depression, fatigue, anxiety, sleepiness, and quality of life. We performed univariable analyses of the relationship between TSPS-phasic and TSPS-tonic, and between each of those measures and the demographic and clinical variables collected at baseline. We then built multivariable linear regression models to find predictors for TSPS-phasic and TSPS-tonic, while including potential confounders and avoiding collinearity.

**Results:**

Fifty-two FM patients were analyzed. 28.85% developed summation during the TSPS-phasic protocol while 21.15% developed summation during the TSPS-tonic protocol. There were no variables associated TSPS phasic or tonic in the univariable analyses and both measures were not correlated. On the multivariate model for the TSPS-phasic protocol, we found a weak association with pain variables. BPI-pain subscale was associated with more temporal summation in the phasic protocol (ß = 0.38, *p* = 0.029), while VAS for pain was associated with less summation in the TSPS-tonic protocol (ß = −0.5, *p* = 0.009).

**Conclusion:**

Our results suggest that, using heat stimuli with pain-60 temperatures, a TSPS-phasic protocol and a TSPS-tonic protocol are not correlated and could index different neural responses in FM subjects. Further studies with larger sample sizes would be needed to elucidate whether such responses could help differentiating subjects with FM into specific phenotypes.

## Introduction

Fibromyalgia is a chronic disease characterized by generalized musculoskeletal pain, fatigue, and cognitive symptoms (1). Despite an unknown etiology (2), research has shown evidence of central nervous system (CNS) involvement, supporting central sensitization and a defective endogenous pain modulation (3, 4). Experimental pai n measurements, such as temporal summation (TS) or Conditioned Pain Modulation (CPM), could contribute to further understanding of the pathophysiology of the disease and its connection to the CNS.

Conditioned Pain Modulation, which is based on the “pain inhibits pain” paradigm (5), consists of the application of two noxious stimuli, and the inhibition of pain from the first noxious stimuli by the second stimuli applied in a different area of the body. CPM measures would thus represent the activity of descending inhibitory pathways.

Temporal summation, on the other hand, is believed to be related to endogenous excitatory pain mechanisms and it consists of repeated or continuous administration of noxious stimuli resulting in the amplification of pain perception despite the same intensity of the stimuli (5, 6). Central sensitization is an abnormal state of increased responsiveness of the spinal and supraspinal neurons leading to low-threshold hypersensitivity (7). It may occur in different areas of the nervous system such as the dorsal horn neurons, which are a crucial part of the pain pathways, after repeated tonic stimulation of C-fibers. This stimulus eventually leads to short- and long-lasting impulse discharges in a wide dynamic range and also in the dorsal horn neurons that increase the excitability of the nociceptive system and enhance the sensation of a second pain stimulus (7, 8), this is known as “wind-up” (8). TS is a method that resembles the “wind up” process by applying a painful frequency (>3 Hz) and stimulating the unmyelinated C fibers (3, 9), and provides information regarding the low pain threshold in fibromyalgia patients and its connection to the CNS (8). TS, therefore, is believed to act as a marker of central sensitization mechanisms.

Temporal summation protocols can be elicited through continuous (tonic), or repetitive (phasic) painful stimuli (10). The evidence regarding the agreement of these two different types of stimuli in individuals with fibromyalgia is scarce, with only a few randomized controlled trials available (10–12). Moreover, the results of these RCTs are conflicting and do not completely identify the TS profile of these individuals (10, 12). It is particularly important to identify which TS paradigms better contribute to the understanding of the central mechanisms behind fibromyalgia pain, considering how important these CNS modulation systems impact the pain felt by fibromyalgia patients (11). Moreover, TS can be evoked by different mechanical, heat, or electrical stimuli (13). Thus, several stimuli can be used in TS protocols to test said phenomenon, creating heterogeneous methodologies of application (14–17). This methodological variety regarding the type of stimuli, number of pulses, and duration can yield distinct results and lead to ambiguous conclusions regarding the TS phenomenon in individuals with chronic pain, especially fibromyalgia (3). A recent, inconclusive systematic review on TS as an endogenous pain modulation marker in individuals with fibromyalgia, attributed its inconclusive results to the flexible and heterogeneous protocols present in the fibromyalgia studies (3). Therefore, this variability in the methodology supports the need for standardized processes to measure TS in chronic pain conditions.

In this study we collected baseline data from fibromyalgia patients including experimental pain measurements including two paradigms of temporal summation: phasic and tonic. In this study, we aimed to explore (i) the relationships between the two protocols of Temporal Summation measures (phasic and tonic) collected and (ii) the associations between these measures and the other clinical variables collected at baseline.

## Materials and Methods

### Study Design and Data Collection

This study is a cross-sectional analysis of our ongoing randomized, double-blind clinical trial NCT03371225. Data for this study were collected from May 13, 2019 to January 22, 2022 from the baseline visits, before the intervention period. This study obeys the Declaration of Helsinki and was approved by the Institutional Review Board of Mass General Brigham's ethics committee under protocol number 2017P002524. All participants have given their written informed consent. A detailed description of the protocol is published elsewhere (18).

Patients between 18 and 65 years old were eligible to participate if they met the diagnosis of fibromyalgia pain according to the American College of Rheumatology (ACR) 2010 criteria and widespread pain for more than 6 months with an average of at least 4 on a 0–10 Visual Analog Scale (VAS) scale without another comorbid chronic pain diagnosis; also patients had to be pain resistant to common analgesics. The exclusion criteria were: the presence of any clinically significant or unstable medical or psychiatric disorder; history of substance abuse within the past 6 months; previous significant neurological history resulting in neurological deficits; severe depression, pregnancy or current opiate use in large doses (more than 30 mg of oxycodone/hydrocodone or equivalent).

### Temporal Summation Paradigms and Questionnaires

#### Temporal Slow Pain Summation (TSPS-phasic)

We first trained subjects to determine the pain-60 test temperature [the temperature inducing pain sensation at a magnitude of 60 on a 0–100 numerical pain scale (NPS)] by applying a Peltier thermode (Medoc Advanced Medical Systems, Ramat Yishai, Israel) on the right forearm using a 30 mm ×30 mm embedded heat pain (HP) thermode. We delivered three short heat stimuli (43, 44, and 45°C), each lasting 7 s. They were asked to report their subjective levels of pain intensity using the visual analog scale for pain (VAS) on a scale ranging from 0, denoting “no pain,” to 100, denoting “the worst pain imaginable.” If the first temperature of 43°C was considered too painful (>60/100), we stopped the series and provided additional stimuli at lower temperatures of 41°C and 42°C. If the three temperatures (43, 44, and 45°C) were unable to achieve the pain-60, we delivered additional stimuli at 46, 47, and 48°C until reaching the desired pain level of 60/100; in the unlikely event that none of those temperatures elicited pain-60, we considered it to be 48°C. We then delivered pulses with rise/fall of 1–2 s, with a rate of change of 8 degrees per second and a delta of 7 degrees, from adapting temperatures to peak temperatures (pain-60), with a plateau of 0.7 s. They received one train of 15 repetitive heat stimuli at 0.4 Hz and pain ratings were asked after the 1st and 15th stimuli: TSPS-phasic was calculated as the difference between those ratings (after the 15th minus after the 1st).

#### Tonic Heat Test Stimulus- (TSPS-tonic)

In addition to the Temporal Summation resulting from the short repetitive stimuli described above, we also calculated the pain summation resulting from the tonic heat test stimulus performed during our Conditioned Pain Modulation paradigm. Ten minutes after the TSPS protocol, we administered the tonic heat test stimulus at the pain-60 temperature continuously for 30 s, and we asked the subjects to rate their pain levels using the visual analog scale for pain (VAS) on a scale ranging from 0, denoting “no pain,” to 100, denoting “the worst pain” imaginable after 10, 20 and 30 s. TSPS-tonic was calculated as the difference between the ratings after 30 s minus the ratings after 10 s.

#### Questionnaires

We collected baseline demographic data and questionnaires including: pain intensities, self-reported depression, anxiety, stress, and sleepiness levels with the visual analog scale-−0–10 point scale [VAS pain (19), VAS depression (20), VAS anxiety (21), VAS stress (20), and VAS sleepiness (19), respectively]; Modified Brief Pain Inventory-BPI (with subscales of average pain ratings and ratings of pain interference in daily living (BPI-pain and BPI-interference, respectively), as well as number of locations in the body with pain (BPI- Number of locations): this scale provides information on various dimensions of pain including how pain developed, the types of pain a patient experiences, time of day pain is experienced, as well as current ways of alleviating pain and the distribution of pain (19, 22); Revised Fibromyalgia Impact Questionnaire (FIQR) (19), this is a 21-item (0–100 points), multiple-choice questionnaire that assess function, overall impact and symptoms (23); quality of life assessed by Quality of Life Scale (QOL), this is a 16-item (16–112 points), multi-purpose questionnaire that yields a profile of functional health and wellbeing scores (24); Patient Reported Outcomes Measurement Information System for anxiety, fatigue and pain (PROMIS anxiety, PROMIS fatigue, and PROMIS pain, 0–5 points respectively) (19); Pittsburgh Sleep Quality Index (25), this questionnaire assesses seven components of sleep quality: subjective sleep quality, sleep latency, sleep duration, habitual sleep efficiency, sleep disturbances, use of sleeping medications, and daytime dysfunction over the last month and a total sum of “5” or greater indicates a “poor” sleeper; and Beck Depression Inventory (0–63 points) (26).

### Statistical Analysis

Descriptive statistics, i.e., mean, frequencies, and percentages, were analyzed to describe the demographical, social, and clinical characteristics of the study participants. We initially performed univariable analyses of the relationship between TSPS-phasic and TSPS-tonic (Pearson correlation), and subsequently between each of those measures and the demographic and clinical variables collected at baseline (univariable linear regressions). We then built multivariable linear regression models with the objective of finding variables associated with TSPS-phasic and TSPS-tonic, while including potential confounders and avoiding collinearity. Model-building followed a purposeful selection procedure (27): we initially included variables with *p* < 0.25 (from the univariable analysis), and tested the other variables by including them in the model one-by-one and, if they became significant in the multivariable model, or if they changed the Beta-coefficients by more than 20%, we kept them in the final model. We also included, based on prior knowledge, the variables age and sex in our models and tested them the same way we tested the other variables: if the variables, once inserted in the multivariable model, became not significant and did not change the Beta-coefficients by more than 20%, they were removed from the model. All statistical analyses and graphical outputs for this paper were generated using SAS software version 9.4 (SAS Institute, Cary NC).

## Results

We included 52 fibromyalgia patients, 86.5% of them female, with a mean of 48.1 years (SD 11.1) in our analysis. The average of VAS pain was 6 (SD 1.77) and a mean of 10 years of fibromyalgia pain (SD 8.6) (Table 1).

**Table 1:** Baseline characteristics of the sample.

**Variables**	**Overall**	**Phasic protocol**		**Tonic protocol**	
	***N* = 52^**1**^**	**Summation,** ***N* = 15^**1**^**	**No summation, *N* = 41^**1**^**	**Summation, *N* = 11^**1**^**	**No summation, N = 41^**1**^**
Age	48.08 (11.12)	46.93 (10.65)	48.54 (11.42)	49.36 (11.62)	47.73 (11.11)
Female	45/52 (87%)	12/15 (80%)	33/37 (89%)	10/11 (91%)	35/41 (85%)
BMI	28.18 (6.86)	30.97 (7.56)	27.05 (6.32)	26.95 (6.11)	28.51 (7.08)
Duration of fibromyalgia	10.38 (8.60)	7.70 (7.06)	11.47 (9.00)	11.45 (10.93)	10.10 (8.00)
Patients in low dose opioids	5/52 (9.6%)	1/15 (6.7%)	4/37 (11%)	1/11 (9.1%)	4/41 (9.8%)
CPM	−0.89 (1.33)	−1.27 (1.08)	−0.74 (1.41)	−0.74 (1.31)	−0.93 (1.35)
Vas pain (0–10)	6.00 (1.77)	5.91 (1.79)	6.04 (1.78)	5.16 (1.60)	6.22 (1.76)
Vas anxiety (0–10)	4.41 (2.64)	3.91 (2.60)	4.61 (2.66)	4.64 (2.53)	4.35 (2.69)
Vas depression (0–10)	3.84 (2.91)	3.33 (2.90)	4.05 (2.92)	2.97 (2.98)	4.08 (2.88)
Vas sleep (0–10)	6.04 (2.57)	5.01 (2.79)	6.45 (2.39)	5.95 (2.91)	6.06 (2.51)
Vas stress (0–10)	5.41 (2.93)	4.55 (3.30)	5.76 (2.73)	4.45 (3.18)	5.67 (2.84)
BPI pain intensity	5.38 (1.58)	5.28 (1.80)	5.41 (1.51)	5.09 (1.54)	5.45 (1.61)
BPI pain interference	5.46 (2.16)	5.15 (2.02)	5.58 (2.23)	4.70 (1.81)	5.66 (2.22)
BPI number of locations with pain (0–72)	12.21 (8.13)	10.53 (7.48)	12.89 (8.39)	11.27 (7.21)	12.46 (8.43)
FIQR (0–100)	55.67 (18.62)	53.81 (22.56)	56.42 (17.07)	49.36 (19.50)	57.36 (18.26)
QOLD (16–112)	68.52 (14.20)	71.60 (19.01)	67.27 (11.81)	70.55 (16.35)	67.98 (13.74)
BDI (0–63)	16.87 (9.18)	16.60 (11.38)	16.97 (8.30)	18.36 (8.03)	16.46 (9.51)
PROMIS anxiety (0–5)	2.66 (0.95)	2.50 (0.90)	2.73 (0.98)	2.66 (1.16)	2.66 (0.91)
PROMIS fatigue (0–5)	3.89 (0.77)	3.38 (0.75)	4.10 (0.69)	3.89 (0.88)	3.90 (0.75)
PROMIS pain (0–5)	3.61 (0.97)	3.43 (1.04)	3.68 (0.95)	3.16 (1.01)	3.73 (0.94)
PSQI (0–21)	12.08 (4.52)	11.20 (4.68)	12.43 (4.46)	11.73 (5.42)	12.17 (4.31)

^1^Mean(SD); n/N (%). QOLS, Quality of Life scale; PROMIS, Patient-Reported Outcomes Measurement Information System; VAS, Visual Analog Scale; PSQI, Pittsburgh Sleep Quality Index; FIQR, Revised Fibromyalgia Impact Questionnaire; BPI, Beck's Depression Inventory; BDI, Brief Depression Inventory. *Races reported as white vs. non-white and Hispanic vs. non-Hispanic, collapsed to increase statistical power. ^†^Presence indicates any increase in pain with continuous or repetitive stimuli; absence indicates no increase or decrease in pain with continuous or repetitive stimuli.

### TSPS – Phasic Protocol

28.85% developed summation during the TSPS-phasic protocol (pain ratings at the end higher than pain ratings at the beginning of the test), 32.69% had no changes during the TSPS-phasic protocol and 38.46% decreased their pain ratings (Table 2). From the univariate models, there were no significantly associated variables with an increase in temporal summation (higher VAS after the stimulus) at a 0.05 significance level (Table 3, Figure S1). Following our multivariable building process, we first included the variables female, age, duration of disease, sleepiness measured by VAS, and PROMIS for fatigue in our model, resulting in an adjusted *R*-square of 0.11. For the next step, we removed the variables with a *p* < 0.1 and that did not change the beta-coefficients of the other variables by more than 20%, resulting in a model with duration of disease and sleepiness measured by VAS and an adjusted *R*-square of 0.12. We then included, one by one, the other variables, keeping them if they fulfilled the previous criteria, resulting in a final multivariate model for the TSPS-phasic protocol, where the BPI-pain subscale was associated with more temporal summation (ß = 0.38, *p* = 0.029), adjusted by pain-60, the duration of fibromyalgia and VAS sleepiness, with an *R*-square of 0.18 (Table 4).

**Table 2:** Temporal summation characteristics of the sample.

**Variables**	**Overall**	**Phasic protocol**		**Tonic protocol**	
	***N* = 52^**1**^**	**Summation, *N* = 15^**1**^**	**No summation, *N* = 41^**1**^**	**Summation, *N* = 11^**1**^**	**No summation, *N* = 41^**1**^**
**Phasic protocol** (dichotomized)- Summation	15/52 (29%)			5/11 (45%)	10/41 (24%)
Phasic protocol (absolute value)	−0.45(1.79)	1.43 (0.78)	−1.22 (1.49)	−0.18 (1.83)	−0.52 (1.80)
1st pain rating	4.04 (2.21)	3.33 (1.8)	4.32 (2.36)	3.82 (1.40)	4.10 (2.40)
Last pain rating	3.59 (2.24)	4.77 (2.08)	3.11 (2.14)	3.64 (2.54)	3.57 (2.18)
**Tonic protocol** (dichotomized)- Summation	11/52 (21%)	5/15 (33%)	6/37 (16%)		
Tonic protocol (absolute value)	−0.88 (2.05)	−0.67 (2.06)	−0.97 (2.06)	1.73 (1.01)	−1.59 (1.64)
1st pain rating	5.94 (1.75)	6.47 (1.13)	5.73 (1.92)	5.09 (2.17)	6.17 (1.58)
Last pain rating	5.06 (2)	5.80 (2.18)	4.76 (1.88)	6.82 (1.60)	4.59 (1.84)
Pain-60 (41^−^48^o^C)
41	2/52 (3.8%)	0/15 (0%)	2/37 (5.4%)	0/11 (0%)	2/41 (4.9%)
43	1/52 (1.9%)	0/15 (0%)	1/37 (2.7%)	0/11 (0%)	1/41 (2.4%)
44	3/52 (5.8%)	1/15 (6.7%)	2/37 (5.4%)	1/11 (9.1%)	2/41 (4.9%)
45	4/52 (7.7%)	1/15 (6.7%)	3/37 (8.1%)	0/11 (0%)	4/41 (9.8%)
46	11/52 (21%)	3/15 (20%)	8/37 (22%)	1/11 (9.1%)	10/41 (24%)
47	13/52 (25%)	7/15 (47%)	6/37 (16%)	4/11 (36%)	9/41 (22%)
48	18/52 (35%)	3/15 (20%)	15/37 (41%)	5/11 (45%)	13/41 (32%)
Mean	46+5 (1.7)	46.7 (1.1)	46.43 (1.9)	47.1 (1.2)	46.3 (1.8)

*^1^Mean(SD); n/N (%)*.

**Table 3:** Univariable analysis of the association between TSPS-phasic and TSPS-tonic with other baseline characteristics.

**Variable**	**TSPS-phasic**		**TSPS-tonic**	
	**Correlation coefficient^**1**^**	***p*-Value**	**Correlation coefficient^**1**^**	***p*-Value**
TSPS-tonic	0.108	0.445	1.00	–
TSPS-phasic	1.00	–	0.108	0.445
Age	−0.249	0.075	−0.049	0.732
BDI	0.019	0.894	0.024	0.867
BMI	0.073	0.605	−0.020	0.686
Opioid use	−0.06	0.652	−0.01	0.89
BPI pain	0.147	0.298	−0.057	0.686
BPI interference	−0.020	0.887	−0.115	0.418
BPI number of locations with pain	0.1	0.477	0.18	0.199
CPM	−0.973	0.492	0.085	0.547
Duration	−0.330	0.017	0.055	0.696
FIQR	0.035	0.804	0.007	0.960
Pain-60	0.102	0.474	0.164	0.246
PSQI	−0.080	0.571	0.035	0.805
QOLS	0.032	0.823	−0.208	0.139
VAS pain	0.064	0.651	−0.209	0.137
VAS anxiety	0.071	0.618	0.116	0.413
VAS depression	0.057	0.688	−0.045	0.753
VAS sleepiness	−0.199	0.158	0.062	0.661
VAS stress	−0.043	0.761	−0.129	0.362
PROMIS anxiety	0.071	0.616	−0.022	0.875
PROMIS fatigue	−0.191	0.175	0.141	0.318
PROMIS pain	0.066	0.643	−0.090	0.524
**Variable**	**Mean difference** ^**2**^	***p*** **-Value**	**Mean difference** ^**2**^	***p*** **-Value**
Female	−1.265	0.082	−0.362	0.668
White	0.255	0.646	0.721	0.251
Hispanic	0.188	0.769	−0.514	0.480

*^1^Pearson correlation.*

*^2^Unpaired t-test.*

*PROMIS, Patient-Reported Outcomes Measurement Information System; VAS, Visual Analog Scale; PSQI, Pittsburgh Sleep Quality Index; FIQR, Revised Fibromyalgia Impact Questionnaire; BPI, Beck's Depression Inventory; BDI, Brief Depression Inventory; QOLS, Quality of Life Scale*.

**Table 4:** TSPS-phasic multivariate linear model.

**Variables**	**Beta coefficient**	**Standard error**	**95% CI**	***p*-Value**
BPI (pain)	0.38	0.167	0.04, 0.71	0.029*
Temperature to elicit pain 60/100 (Pain-60)	0.17	0.138	−0.12, 0.45	0.223
Duration	−0.07	0.026	−0.12, −0.01	0.013*
Sleepiness (VAS)	−0.26	0.1	−0.46, −0.06	0.014*

^*^Significant p-values.

### TSPS – Tonic Protocol

21.15% developed summation during the TSPS-tonic protocol. 25% had no changes in the TSPS-tonic protocol and 53.85% decreased their pain ratings (Table 2). From the univariate models, there was no significantly associated variables with an increase in temporal summation (higher VAS after the stimulus) at a 0.05 significance level (Table 3, Figure S1). Following our multivariable building process, we first included the variables female, age, pain measured by VAS, QOLs, pain 60, and number of locations with pain in our model, resulting in an adjusted *R*-square of 0.07. For the next step, we removed the variables with a *p* < 0.1 and that did not change the beta-coefficients of the other variables by more than 20%, resulting in a model with pain measured by VAS, QOLs and number of locations with pain and an adjusted *R*-square of 0.10. We then included, one by one, the other variables, keeping them if they fulfilled the previous criteria, resulting in a final multivariate model for the TSPS-tonic protocol with VAS pain was associated with less temporal summation (ß = −0.45, *p* = 0.019), adjusted by QOL, number of locations in the body with pain from the BPI, and VAS anxiety, with an adjusted *R*-square of 0.13 (Table 5).

**Table 5:** TSPS-tonic multivariate linear model.

**Variables**	**Beta coefficient**	**Standard error**	**95% CI**	***p*-Value**
Pain (VAS)	−0.5	0.184	−0.87, −0.13	0.009*
QOLS	−0.02	0.02	−0.06, 0.02	0.233
Anxiety (VAS)	0.21	0.129	−0.04, 0.47	0.104
BPI number of pain locations	0.06	0.33	−0.01, 0.12	0.103

QOLS, Quality of Life Scale; VAS, Visual Analog Scale. *Significant p-value.

## Discussion

Our study aimed to explore the phenomenon of temporal summation in fibromyalgia patients. We explored the agreement between phasic and tonic temporal summation protocols, as well as the relationship with patients' characteristics. Temporal summation of pain happened in only a minority of our sample and both protocols did not correlate. In our exploratory analysis using multivariable linear regression modeling, we found that the relationship between baseline covariates and heat temporal summation could be different for the two protocols.

Fibromyalgia is a complex and heterogeneous syndrome. In fact, this challenging diagnosis has been a matter of debate for decades, relying solely on symptom evaluation (18), although researchers have attempted to identify biomarkers and surrogates to support it (28). The frequently replicated but still contradictory finding of enhanced temporal summation of pain in FM has been quite persuasive and has led to an increase of research on the central sensitization paradigm in FM. In our study, only a minority of our FM sample in fact showed increased temporal summation, regardless of the protocol (tonic or phasic), a result that comes to add to other similar ones in the literature (10). The differences found in our results could be the consequence of the characteristics of the clinical condition and also our study sample, as it has contrasts in some features from the overall fibromyalgia population. Given our protocol, FM patients included should have been willing and be capable to perform 30 min of aerobic exercise, have less than mild to moderate depression levels (BDI <30) and be able to commit to multiple in-person visits throughout our parent RCT [see our published protocol for a detailed description of the study visits (29)]. There is evidence of exercise improving chronic pain including pain thresholds but is still contradictory if an exercise program can induce changes in central sensitization in chronic pain patients (30, 31). Also, different studies have shown prominent prevalence of Major Depressive Disorder diagnosis and moderate-to-severe depressive symptoms in FM (32–34), which have been linked to the severity of the disease (35); Uhl et al. (36) found a positive correlation of patients with major depression with an increase pain perception after frequent noxious stimuli in a temporal summation protocol. Depression is a key factor for the epidemiology and pathophysiology of FM, while they might even share similar genetic pathways (37). Depression is a factor that might moderately modify pain-related negative affect, therefore creating variance in pain intensity within the population (38). There are similar changes in neuroplasticity between patients with chronic pain and depression (39, 40). Therefore, our sample of patients who were capable and willing to do exercise and were not depressed, could have less summation than other samples of FM patients.

Our study did not find a correlation between the phasic and the tonic TSPS protocols. There is heterogeneity in the description of protocols for temporal summation in the literature. O'Brien et al. (3) published a review of TS in fibromyalgia patients. They assessed 23 studies (*n* = 648) and found a large variability regarding population, methodology and parameters of stimulations (type of stimuli, number of stimuli, duration, and location): the most common stimuli used was thermal, followed by mechanical and electrical. Staud et al. (41) found significant differences in the pain ratings of the fifth stimulus in FM patients, but no difference between the 1st and last stimulus between groups: they studied 14 FM patients and 19 healthy controls using a phasic TS protocol over the palmar surface of the hands with a heat stimuli. Moreover, Potvin et al. (10) included 72 fibromyalgia patients and 39 healthy women and tested tonic heat temporal summation over the left arm: there was no significant difference between healthy controls and fibromyalgia patients, but they found that patients with a temperature of ≥45°C as pain-50 had an increased summation of pain after the stimulus compared to patients with pain-50 <45°C temperature. The phasic protocol for temporal summation is the most reported in the literature (9, 15, 42–46). For example, Granot et al. (15) compared the phasic and tonic protocols of temporal summation in healthy subjects and found a significant correlation, suggesting that both represent similar neurophysiological processes. Also, Granot et al. (47) aimed to measure the psychophysics of the phasic and tonic phases in healthy volunteers with a different protocol and found a correlation between the tonic and phasic protocols, but no differences in the pain scores. However, most of the research describing the difference between protocols are in healthy subjects.

Given the differences between the tonic and phasic TSPS we found in our results, which may rely on several aspects, we believe that these two types of temporal summation measures could be, in fact, representing different phenomena in chronic pain patients. One possible explanation for the discrepancy between the two paradigms in our study could be that the pain-60 used might have been too high, in particular for the tonic paradigm, curtailing the subject's ability to discern differences in pain ratings. The average pain rating after the first stimulus in the phasic protocol was, on a scale from 0 to 10, 4.04 (SD 2.21) and after the last stimulus 3.59 (SD 2.24). These measures were higher during the tonic paradigm, with average pain rating after the first recorded measure of 5.94 (SD 1.75) and after the last measure 5.06 (SD 2.00). This fact can also explain why only 28.9% of our subjects developed summation during the phasic protocol and 21.2% during the tonic protocol. This hypothesis is supported by the fact that in the subgroup with pain-60 at 48 degrees Celsius (who possibly did not feel the same pain levels as the other patients, since 48 degrees was our maximum temperature), the incidence of temporal summation with the phasic protocol was less than the overall, 16.7%, and with the tonic protocol it was more than the overall, 27.8%.

The mechanisms underlying the temporal summation phenomenon present in some FM patients are still not well understood. It is believed that temporal summation of pain happens in C-fibers located at the spinal dorsal-horn neurons, mediated by glutaminergic excitatory synapses and thus NMDA receptors, since the blockade of NMDA receptors has been associated with decrease in temporal summation to nociceptive stimuli (48, 49). Staud et al. (50) found that, although FM subjects did have more temporal summation than healthy controls at first, they did not respond differently to external NMDA-blockage. This suggests that differences in responsiveness in these pathways could not explain entirely the maladaptive pain processing in FM. Instead, alternative pain modulation mechanisms, including disruptions in inhibitory descendent control and emotional-cognitive circuitry, can play equally important roles in different subsets of FM patients. One of the possible explanations would be that temporal summation of pain is a “trait” of a subset of patients rather than a “state” in FM. It is worth mentioning that phasic temporal summation protocols could also be testing phenomena known as offset analgesia (OA) and onset hyperalgesia (OH). The paradigms for testing OA and OH require transient increases or decreases in thermal noxious stimuli, and are believed to be related to time-dependent anticipation of pain relief with a decreasing temperature or anticipation of pain worsening with increasing temperature. These responses to expectation/anticipation would be thus associated with central, rather than peripheral, nociceptive pathways (51).

Central sensitization phenotypes, as measured by temporal summation, are not homogenous across different strata of FM patients. The attempt to find FM subsamples, sharing similar syndromic and pathological mosaics, has been suggested before and is potentially the future fostering multi-modal and individually-tailored treatment in FM (1). This seems likely to be an adequate pathway to better understand the complexity of biomarkers, prognosis, and treatment response in FM, considering the complexity of this widespread pain entity. Since our sample of patients who developed summation in the phasic and tonic protocols do not overlap (only five subjects developed summation in both protocols), the use of different paradigms could also help in identifying these subsamples who would potentially respond differently to therapies.

In our exploratory analysis with multivariate models, which were not adjusted for multiple comparisons, pain scales measured with the BPI-pain subscale and VAS were found to be associated with the phasic-TSPS and tonic-TSPS, respectively. Interestingly, age and sex were not associated with temporal summation in any of the final multivariable models (52, 53). In the phasic-TSPS protocol, more pain in the BPI scale was associated with higher temporal summation, when adjusted for the duration of fibromyalgia, pain-60 levels, and sleepiness measured by VAS. In accordance, in different chronic pain populations, more temporal summation has been associated with a poorer prognosis of pain progression (54), worse disease severity (55–57), and higher experimental and clinical pain (56–59). In contrast, the relationship between tonic TSPS and pain levels was inverse, with higher pain being associated with less summation, when adjusted for QOLs, number of locations of the body with pain, and anxiety measured by VAS. This result was unexpected, but at the same time, very interesting. It is worth mentioning that this relationship, albeit maintaining the same directionality as in the univariable analysis, only became significant after the inclusion of the other variables in the model. We speculate that one possible explanantion could be that higher pain levels in the VAS could be related to more activation of emotional affective circuits, as seen in previous studies (60–64). Thus, one plausible explanation for the unexpected finding of an inverse relationship between pain levels and tonic pain summation is an overactivation of emotional circuits in subjects with higher baseline VAS, leading to worse anticipation of pain and leading to higher ratings of pain at the initial of the tonic protocol that attenuates in the second and third trials. This is supported by the fact that the average first pain rating during the tonic protocol for individuals that developed summation was 5.09 (SD 2.17) and for those who did not develop summation was 6.17 (SD 1.58) (Table 2).

In addition, regarding the tonic summation protocol, the number of locations in the body with pain, a variable that is possibly related to pain distribution, confounded the relationship between pain measured with VAS and TSPS tonic. When the variable with the number of locations was introduced in the multivariable model, the effect of VAS-pain in TSPS was even more negative, that is, the higher the VAS-pain, the less tonic summation. Interestingly, the associations between tonic-TSPS and pain distribution, and tonic-TSPS and anxiety were positive, meaning that the higher the number of pain locations and the higher the anxiety scores (measured by VAS) resulted in higher tonic-TSPS. These relationships should be interpreted with caution given our relatively small sample size and the fact that they were not signitificant at a significance level of 0.05.

One of the limitations of our study is that we did not measure pain catastrophizing. Pain catastrophizing has been overly associated with other pain-related outcomes in fibromyalgia, even as an independent agent in pain processing mechanisms (65). Kim et al. (66) showed a significant moderate correlation between tonic temporal summation and scores on the Pain Catastrophizing Scale (PCS) in fibromyalgia patients (*r* = 0.53, *p* < 0.05). In the same way, catastrophizing has also shown positive correlations to temporal summation in healthy samples (15, 67, 68) and in other chronic pain populations (69–71). Hasset et al. (72) also found a strong correlation (0.71) between pain catastrophizing and depression and since we excluded from our sample individuals with BDI >30, we might have introduced selection bias, thus excluding individuals with high levels of pain catastrophizing who would be more prone to develop pain summation. Indeed, VAS scale may reflect more the emotional aspects of pain severity than central sensitization.

In addition to the association found between the BPI-pain assessments and phasic-TSPS, the duration of fibromyalgia and sleepiness were considered confounders in the multivariate model. These variables were inversely related to the outcome; patients with longer disease duration and more significant VAS sleepiness showed less phasic pain summation. Again, one possible explanation could be that the association between these variables and other mechanisms related to pain perception, such as attention and coping strategies, could contribute to this finding. In a prospective cohort (73), patients with a more substantial period since diagnosis develop ameliorated emotional coping strategies than individuals recently diagnosed. Regarding the negative correlation between the phasic temporal summation of pain and sleepiness, attentional circuitry of pain processing and basal autonomic activity were likely involved, as these supraspinal networks are intrinsically related to the magnitude of pain perception (74). Sleepiness is related to an individual's intrinsic attention (75). Adams et al. suggest that the degree to which an individual perceives pain is associated with their level of awareness (75). Moreover, increased sleepiness could signify a greater overall emotional relaxation of the patient, considering that when sleeping, the parasympathetic nervous system is dominant compared to the sympathetic nervous system (76). Since increased sympathetic nervous system activity is associated with increased pain perception (77), it is also possible that a decreased arousal in our sample with higher VAS sleepiness scores could be affecting the neurobiological circuitry involved in the summation of pain in these individuals. Temporal summation is dependent on biological processes that involve glutamatergic excitatory receptors in the central nervous system (3); therefore, this decrease in the excitatory arousal could be lowering the activity of this circuitry, decreasing phasic TSPS scores.

Another limitation in our study was the fact we did not ask our subjects the maximum pain levels felt during our summation paradigms. Therefore, they might have developed summation during our tests but we were not able to capture it at the pre-defined timepoints because the pain levels decreased afterwards. Another limitation is the fact that, although the surface temperature of the skin was measured by the thermode, and we know that such superficial temperature behaved as we planned, the same is not necessarily true for the temperature in the subcutaneous tissue. This might be problematic in particular for the tonic stimulus, when the dynamics of tissue perfusion and heat dissipation could have played a role in the absence of tonic summation observed in most of our subjects. Another limitation is the fact that both our protocols for summation involved response to heat, therefore, our findings could be different if other stimuli, i.e., pressure, were used (78). Finally, due to the exploratory nature of our analyses, no corrections for multiple comparisons were made. Thus, the results from our univariable and, in particular, our multivariable models should be interpreted as hypothesis- generating and, by no means, confirmatory. Further studies with larger sample sizes would be needed to elucidate the relationship between clinical variables and different temporal summation protocols in chronic pain patients.

## Conclusion

Temporal summation is believed to be a tool for the measurement of central sensitization by applying continuous or intermittent noxious stimulus and measuring the pain perception. From the 52 fibromyalgia patients included in our study less than 30% presented temporal summation with heat stimuli based on pain-60 temperatures. Heat phasic and tonic TSPS were also not correlated, and could potentially be used as markers differentiating FM into specific phenotypes. However, future studies are needed to standardize the tonic and phasic temporal summation protocols, and larger sample sizes could undercover the differences between these temporal summation protocols and their relationship to clinical variables in FM and other chronic pain patients.

## Data Availability Statement

The raw data supporting the conclusions of this article will be made available by the authors, without undue reservation.

## Ethics Statement

The studies involving human participants were reviewed and approved by Institutional Review Board of Mass General Brigham. The patients/participants provided their written informed consent to participate in this study.

## Author Contributions

LC-B and AC-R contributed to the design of the study, the acquisition, analysis, and interpretation of data, and drafted the paper. WC, GR, KM, and FF contributed to design of the study and revised the paper critically for important intellectual content. IR-S, KP-B, PG-M, PM, PT, PC, JP, AM, and KV-A, contributed to data acquisition and interpretation and drafted the paper. All authors approved final version to be published and agree to be accountable for all aspects of the work in ensuring that questions related to the accuracy or integrity of any part of the work are appropriately investigated and resolved.

## Funding

This work was supported by NIH under grant (1R01AT009491-01A1).

## Conflict of Interest

The authors declare that the research was conducted in the absence of any commercial or financial relationships that could be construed as a potential conflict of interest.

## Publisher's Note

All claims expressed in this article are solely those of the authors and do not necessarily represent those of their affiliated organizations, or those of the publisher, the editors and the reviewers. Any product that may be evaluated in this article, or claim that may be made by its manufacturer, is not guaranteed or endorsed by the publisher.
