# Supplementary figures and images for: Temporal Summation in Fibromyalgia Patients: Comparing Phasic and Tonic Paradigms
Source: Front Pain Res (Lausanne). 2022 Jun 22;3:881543. doi: 10.3389/fpain.2022.881543 (PMC9261961; doi:10.3389/fpain.2022.881543)

Supplementary Figure 1.

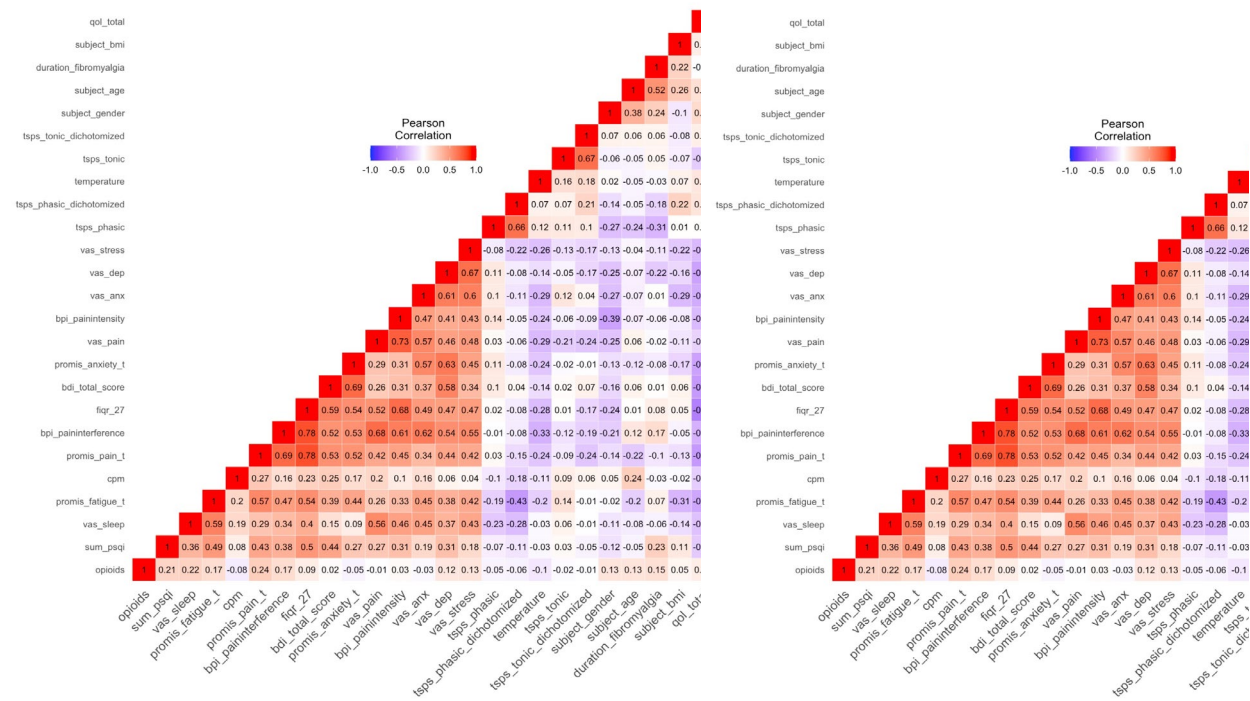

Supplement: Supplementary file 1 [file Image_1.pdf]
